# Supplementary material for: Brain-behaviour modes of covariation in healthy and clinically depressed young people
Source: Sci Rep. 2019 Aug 8;9:11536. doi: 10.1038/s41598-019-47277-3 (PMC6687746; doi:10.1038/s41598-019-47277-3)
Supplement: Supplementary file 1 — Supplementary Information [file 41598_2019_47277_MOESM1_ESM.pdf]

Supplementary Information

Brain-behaviour modes of covariation in healthy and clinically depressed young people

Agoston Mihalik, Fabio S. Ferreira, Maria J. Rosa, Michael Moutoussis, Gabriel Ziegler, Joao M. Monteiro, Liana Portugal, Rick A. Adams, Rafael Romero-Garcia, Petra E. Vértes, Manfred G. Kitzbichler, František Váša, Matilde M. Vaghi, Edward T. Bullmore, Peter Fonagy, Ian M. Goodyer, Peter B. Jones, NSPN Consortium, Raymond Dolan, Janaina Mourão-Miranda

Methods

Self-report questionnaires

- Antisocial Behaviours Checklist (ABQ)[1] – self-report questionnaire for symptoms of antisocial behaviour based on DSM-IV conduct disorder items. The questionnaire was designed solely for the purpose of the NSPN project (11 items).
- Antisocial Process Screening Device (APSD)[2] – self-report scale measuring psychopathic traits and antisocial behaviour (20 items).
- Barratt Impulsive Scale (BIS)[3] – self-report questionnaire assessing personality and behavioural constructs of impulsiveness (30 items).
- Child and Adolescent Dispositions Scale (CADS)[4] – self-report measure of the three underlying dimensions of cognitive control of behaviour: pro-sociability, negative emotionality and daring (57 items).
- Child Trauma Questionnaire (CTQ)[5] – self-report inventory screening for histories of abuse and neglect, which covers five types of maltreatment: emotional, physical, and sexual abuse, and emotional and physical neglect (28 items).
- Drugs Alcohol and Self-Injury (DASI)[1] – self-report measure assessing the frequency of drug and alcohol use as well as the frequency, methods and motives of non-suicidal self-harm acts. The questionnaire was designed solely for the purpose of the NSPN project (16 items).
- Inventory of Callous-Unemotional Traits (ICU)[6] – self-report inventory of assessing 3 domains of callous and unemotional traits: callousness, uncaring, and unemotional (24 items).
- Kessler Psychological Distress Scale (K10)[7] – self-report measurement of psychological distress (10 items).
- Leyton Obsessional Inventory (LOI)[8] – self-report questionnaire measuring obsessional and anxiety symptoms (11 items).
- Moods and Feelings Questionnaire (MFQ)[9] – self-report questionnaire measuring depressive symptoms in the last 2 weeks (33 items).
- Revised Children's Manifest Anxiety Scale (RCMAS)[10] – self-report questionnaire measuring anxiety symptoms (28 items).
- Rosenberg Self-Esteem Scale (SES)[11] – self-report questionnaire measuring global self-esteem or feelings of self-worth and self-acceptance (10 items).
- Schizotypal Personality Questionnaire (SPQ)[12] – self-report scale measuring schizotypal personality traits (74 items).

- Wechsler Abbreviated Scale of Intelligence (WASI)[13] – matrix reasoning and vocabulary subsets of the Wechsler Abbreviated Scale of Intelligence (WASI) designed to assess fluid and crystallized intelligence, respectively (2 items).
- Warwick Edinburgh Mental Wellbeing Scale (WEMWBS)[14] – self-report instruments spanning the theoretical distribution of common mental symptoms and wellbeing (14 items).

Socioeconomic deprivation index

The socioeconomic deprivation index is a small-area model-based households in poverty estimate and reflects the proportion of deprived/poor households around the subject's residence (for full details see: https://www.ons.gov.uk/peoplepopulationandcommunity/personalandhouseholdfinances/incomeandwealth/bulletins/smallareamodelbasedhouseholdsinpovertyestimatesenglandandwales/financialyearending2014). The index was calculated by a searchable web page provided by the Office of National Statistics, UK (https://www.ons.gov.uk/), however, we note that the online tool is no longer available, and only a downloadable updated dataset can be found here: https://www.ons.gov.uk/peoplepopulationandcommunity/personalandhouseholdfinances/incomeandwealth/datasets/householdsinpovertyestimatesformiddlelayersuperoutputareasinenglandandwales.

Brain visualization

The glass brains were created using the BrainNet Viewer software[15]. In brief, BrainNet Viewer is a user-friendly MATLAB toolbox that was designed to visualize brain connectomes, as those in Figs 3-4 and Figs S4-5. The nodes were created using the coordinates of the centroids of the previously defined brain regions and the edges represent the brain connections found between the different regions, in which the red ones represent connections positively correlated with the canonical brain variates and the blue ones represent the ones negatively correlated with the canonical brain variates. The thickness of the edges is proportional to the absolute correlation value. The MNI coordinates (x, y, z) of all brain region, their labels, anatomical and functional assignments can be found in Table S7.

Multiple hold-out framework

We also used a multiple hold-out approach for choosing the optimal number of PCA components and estimating the Family Wise Error (FWE) corrected *p*-value on the canonical variates. This approach was implemented based on the framework proposed by Monteiro et. al[16] (Fig. S6):

1. The data matrices $\mathbf{X}_{n\times p}$ and $\mathbf{Y}_{n\times q}$ are randomly split in two different sets of data, an optimisation set (80% of the total number of subjects), $\mathbf{X}_{op}$ and $\mathbf{Y}_{op}$, and a hold-out set (20% of the data), $\mathbf{X}_{ho}$ and $\mathbf{Y}_{ho}$ (Fig. S6a).
2. For each PCA dimensionality d ($d = 5,10,25,50,75,100,150,200$), the optimisation matrices, $\mathbf{X}_{op}$ and $\mathbf{Y}_{op}$, are randomly split 50 times into a training set (80% of the optimisation set), $\mathbf{X}_{op_{tr}}$ and $\mathbf{Y}_{op_{tr}}$, and a testing set (20% of the optimisation set),

$\mathbf{X}_{op_{te}}$ and $\mathbf{Y}_{op_{te}}$. For each random split, the reduction step is performed on the training set, $\mathbf{X}_{op_{tr}}$ and $\mathbf{Y}_{op_{tr}}$, and then CCA is applied to the reduced matrices, $\mathbf{X}^{d}_{op_{tr}}$ and $\mathbf{Y}^{d}_{op_{tr}}$, to obtain the combined PCA+CCA basis vectors $\mathbf{u}_{tr}$ and $\mathbf{v}_{tr}$. The test canonical correlations, $\mathbf{q}_{te} \in \mathbb{R}^{d\times 1}$, are computed by projecting the testing set onto these basis vectors (Fig. S6b). At the end of this procedure, for each PCA dimensionality d, a matrix of test canonical correlations ($\mathbf{Q}_{te} \in \mathbb{R}^{d\times 50}$) is obtained, in which each row corresponds to a CCA component. The optimal number of PCA components is chosen based on the maximal average test canonical correlation of the first CCA components. In other words, the number of PCA components is chosen to maximise the test canonical CCA correlation (i.e. CCA correlation obtained when the test data was projected onto the PCA+CCA basis vectors estimated based on the train data).

3. PCA is applied to $\mathbf{X}_{op}$ and $\mathbf{Y}_{op}$ using the optimal number of components, d^*, resulting in the reduced data matrices $\mathbf{X}_{d^*}$ and $\mathbf{Y}_{d^*}$. $\mathbf{X}_{d^*}$ and $\mathbf{Y}_{d^*}$ are used to compute the combined PCA+CCA basis vectors, $\mathbf{u}_{ho}$ and $\mathbf{v}_{ho}$. The "true" hold-out canonical correlations, $\mathbf{q}_{ho} \in \mathbb{R}^{d^*\times 1}$, are computed by projecting the hold-out set, $\mathbf{X}_{ho}$ and $\mathbf{Y}_{ho}$, onto these basis vectors (Fig. S6c).
4. For assessing the statistical significance of the hold-out canonical correlations, we used permutation tests. First, the rows of $\mathbf{Y}_{d^*}$, are permuted to obtain $\mathbf{Y}^{+}_{d^*}$ then we applied CCA resulting in "permuted" combined PCA+CCA basis vectors $\mathbf{u}^{+}_{ho}$ and $\mathbf{v}^{+}_{ho}$. Third, we projected the hold-out set, $\mathbf{X}_{ho}$and $\mathbf{Y}_{ho}$, onto these basis vectors to obtain "permuted" hold-out canonical correlations $\mathbf{q}^{+}_{ho} \in \mathbb{R}^{d^*\times 1}$. Next, we repeated the permutation procedure 10,000 times resulting in a matrix of "permuted" hold-out canonical correlations $\mathbf{Q}^{+}_{ho} \in \mathbb{R}^{d^*\times 10000}$ (Fig. S6c). For each row of $\mathbf{Q}^{+}_{ho}$ (representing a CCA component), a p-value is computed by assessing the number of times the permuted canonical correlations are equal or higher than the maximal "true" hold-out canonical correlation. We note that in contrast to the canonical correlations of the training set, the hold-out canonical correlations are not ordered. At the end of this procedure, a vector of *p*-values is obtained for each CCA component ($\mathbf{p} \in \mathbb{R}^{d^*\times 1}$). This allows one to estimate the number of significant CCA components accounting for FWE (i.e. any CCA component with $p_{FWE} < 0.05$ is considered statistically significant).
5. Steps 1-4 are repeated 9 more times (10 different hold-out sets in total). The obtained *p*-values for each hold-out set are corrected for multiple comparisons using *Bonferroni* correction (i.e. $\alpha = 0.05/10 = 0.005$), which means that only the hold-out sets with $p_{corr} \leq 0.005$ are considered statistically significant. Finally, the best hold-out set is chosen based on the lowest p_{corr}.

Tables

Table S1: CCA behaviour correlations computed by correlating the behavioural variables and the behavioural canonical variates of the first CCA mode, when using the permutation framework. Top 20 most positively and top 20 most negatively correlated items shown only.

Clinical label	Questionnaire	Correlation
Male	Demographics	0.68
Age	Demographics	0.38
Do you enjoy doing things that are risky or dangerous?	CADS	0.36
I felt I had a number of good qualities	SES	0.35
During the last month, how often did you drink spirits?	DASI	0.35
Do you like TV, movies, comics, or electronic games with a lot of violence in them?	CADS	0.34
During the last month, how often did you drink beer or cider?	DASI	0.34
In the last 6 months, how often have you been drunk in the way described in Q7?	DASI	0.33
I was able to do things as well as most people	SES	0.33
Do you like rough games and sports?	CADS	0.32
Do you feel confident that you can handle life's challenges?	CADS	0.28
I've been able to make up my own mind about things	WEMWBS	0.28
Do you react with little or no emotion to both positive and negative things?	CADS	0.28
Are you calm and easy-going?	CADS	0.28
Are you proud of yourself?	CADS	0.28
I was satisfied with myself	SES	0.28
You tease or make fun of other people	APSD	0.27
Are you energetic when you have a job to do?	CADS	0.26
I felt that I was as good as anyone else	SES	0.26
Crystallised intelligence (vocabulary)	WASI	0.26
I sometimes blamed myself for things that weren't my fault	MFQ	-0.35
At times, I thought I was no good at all	SES	-0.36
Excluding the last month, have you tried to hurt yourself on purpose without trying to kill yourself in the last 12 months?	DASI	-0.36
I did everything wrong	MFQ	-0.36
I worried about what my parents would say to me	RCMAS	-0.36
Excluding the last month, how have you tried to hurt yourself without trying to kill yourself in the last 12 months?	DASI	-0.37
I thought about dying	MFQ	-0.37
My feelings got hurt easily	RCMAS	-0.37
Do you get upset easily?	CADS	-0.37
In the last month, how have you tried to hurt yourself without trying to kill yourself?	DASI	-0.37
I thought about killing myself	MFQ	-0.38
I was afraid of a lot of things	RCMAS	-0.38

I worried what other people thought about me	RCMAS	-0.38
Are you emotional?	CADS	-0.38
Often I felt sick to my stomach	RCMAS	-0.39
In the last month, why have you tried to hurt yourself without trying to kill yourself?	DASI	-0.39
I thought my family would be better off without me	MFQ	-0.44
I cried a lot	MFQ	-0.44
I thought I looked ugly	MFQ	-0.48
Female	Demographics	-0.68

Table S2: CCA connectivity correlations computed by correlating the brain connectivity variables and the brain canonical variates of the first CCA mode, computed using the permutation framework. Top 20 most positively and top 20 most negatively correlated brain connections shown only. **Functional networks:** Default Mode Network (DMN); Dorsal Attention Network (DAN); Frontoparietal Network (FPT); Limbic Network (LMB); Somatomotor Network (SMT); Ventral Attention Network (VAN); Subcortex (SBC); Visual Network (VIS). **Anatomical regions:** [1]Anterior Cingulate and Medial Prefrontal Cortex; [2]Auditory Association Cortex; [3]Basal Ganglia; [4]Dorsal Stream Visual Cortex; [5]Dorsolateral Prefrontal Cortex; [6]Early Auditory Cortex; [7]Early Visual Cortex; [8]Hippocampus; [9]Inferior Frontal Cortex; [10]Inferior Parietal Cortex; [11]Insular and Frontal Opercular Cortex; [12]Lateral Temporal Cortex; [13]MT+ Complex and Neighboring Visual Areas; [14]Medial Temporal Cortex; [15]Orbital and Polar Frontal Cortex; [16]Paracentral Lobular and Mid Cingulate Cortex; [17]Posterior Cingulate Cortex; [18]Posterior Opercular Cortex; [19]Premotor Cortex; [20]Primary Visual Cortex; [21]Somatosensory and Motor Cortex; [22]Superior Parietal Cortex; [23]Temporo-Parieto-Occipital Junction; [24]Thalamus; [25]Ventral Stream Visual Cortex.

Label node A	Label node B	Correlation
Left-Insular-Granular-Complex (SMT)[11]	Left-Ventral-IntraParietal-Complex (DAN)[22]	0.69
Right-Lateral-Area-7P (DAN)[22]	Left-Insular-Granular-Complex (SMT)[11]	0.68
Left-Area-OP2-3/VS (SMT)[18]	Left-Ventral-IntraParietal-Complex (DAN)[22]	0.68
Left-Medial-Belt-Complex (SMT)[6]	Left-Ventral-IntraParietal-Complex (DAN)[22]	0.67
Right-Medial-Area-7A (DAN)[22]	Left-Insular-Granular-Complex (SMT)[11]	0.67
Right-Ventral-IntraParietal-Complex (DAN)[22]	Left-Insular-Granular-Complex (SMT)[11]	0.67
Right-Area-OP2-3/VS (SMT)[18]	Right-Ventral-IntraParietal-Complex (DAN)[22]	0.67
Right-Ventral-IntraParietal-Complex (DAN)[22]	Left-Area-OP2-3/VS (SMT)[18]	0.66
Right-Ventral-IntraParietal-Complex (DAN)[22]	Left-Medial-Belt-Complex (SMT)[6]	0.66
Right-Medial-Area-7P (FPT)[22]	Left-Frontal-Opercular-Area-3 (VAN)[11]	0.66
Right-Insular-Granular-Complex (SMT)[11]	Right-Ventral-IntraParietal-Complex	0.66

	(DAN)[22]	
Right-Lateral-Area-7P (DAN)[22]	Left-Area-PFcm (VAN)[6]	0.66
Right-Medial-Belt-Complex (SMT)[6]	Right-Medial-Area-7A (DAN)[22]	0.66
Right-Ventral-IntraParietal-Complex (DAN)[22]	Left-RetroInsular-Cortex (SMT)[6]	0.66
Left-Area-PFcm (VAN)[6]	Left-Lateral-Area-7P (DAN)[22]	0.66
Right-Medial-Area-7P (FPT)[22]	Left-Area-PFcm (VAN)[6]	0.65
Right-Medial-Belt-Complex (SMT)[6]	Right-Ventral-IntraParietal-Complex (DAN)[22]	0.65
Right-Area-OP2-3/VS (SMT)[18]	Left-Ventral-IntraParietal-Complex (DAN)[22]	0.65
Right-Lateral-Area-7P (DAN)[22]	Left-Area-OP2-3/VS (SMT)[18]	0.65
Left-RetroInsular-Cortex (SMT)[6]	Left-Ventral-IntraParietal-Complex (DAN)[22]	0.65
Right-Area-10v (LMB)[1]	Left-Area-p32 (DMN)[1]	-0.09
Left-Polar-10p (LMB)[15]	Left-Area-anterior-47r (DMN)[9]	-0.09
Left-Polar-10p (LMB)[15]	Left-Area-8Av (DMN)[5]	-0.09
Left-Polar-10p (LMB)[15]	Left-Area-10v (LMB)[1]	-0.10
Right-Area-ventral-23-a+b (DMN)[17]	Left-Area-8Ad (DMN)[5]	-0.10
Right-Area-31pd (DMN)[17]	Left-Parieto-Occipital-Sulcus-Area-2 (DMN)[17]	-0.10
Right-Area-9-Middle (DMN)[1]	Left-Area-p32 (DMN)[1]	-0.10
Right-Area-8Ad (DMN)[5]	Left-Area-PGs (DMN)[10]	-0.10
Right-Area-ventral-23-a+b (DMN)[17]	Left-Area-7m (DMN)[17]	-0.10
Right-Area-p32 (DMN)[1]	Left-Area-ventral-23-a+b (DMN)[17]	-0.10
Left-Polar-10p (LMB)[15]	Left-Area-9-Posterior (DMN)[5]	-0.10
Left-Area-10v (LMB)[1]	Left-Area-p32 (DMN)[1]	-0.10
Left-Polar-10p (LMB)[15]	Left-Area-8C (FPT)[5]	-0.10
Left-Polar-10p (LMB)[15]	Left-Area-8Ad (DMN)[5]	-0.11
Right-Area-10v (LMB)[1]	Left-Polar-10p (LMB)[15]	-0.11
Left-Area-PGs (DMN)[10]	Left-Polar-10p (LMB)[15]	-0.12
Left-Area-PFm-Complex (DMN)[10]	Left-Polar-10p (LMB)[15]	-0.12
Left-Area-posterior-10p (DMN)[15]	Left-Polar-10p (LMB)[15]	-0.12
Left-Area-9-Middle (DMN)[1]	Left-Area-p32 (DMN)[1]	-0.12
Right-Polar-10p (LMB)[15]	Left-Polar-10p (LMB)[15]	-0.13

Table S3: CCA behaviour correlations computed by correlating the behavioural variables and the behavioural canonical variates of the second CCA mode, when using the permutation framework. Top 20 most positively and top 20 most negatively correlated items shown only.

Clinical label	Questionnaire	Correlation
I've been feeling cheerful	WEMWBS	0.38
I've been feeling interested in other people	WEMWBS	0.38
Are you cheerful?	CADS	0.36
Do you enjoy being with other people your age?	CADS	0.33

I've been dealing with problems well	WEMWBS	0.33
I've been able to make up my own mind about things	WEMWBS	0.30
I've been feeling relaxed	WEMWBS	0.30
I've been feeling good about myself	WEMWBS	0.28
I've been feeling confident	WEMWBS	0.27
I've been feeling useful	WEMWBS	0.27
I am happy-go-lucky	BIS	0.27
I've been feeling close to other people	WEMWBS	0.26
Do you feel confident that you can handle life's challenges?	CADS	0.24
I've been thinking clearly	WEMWBS	0.24
I've had energy to spare	WEMWBS	0.23
I've been interested in new things	WEMWBS	0.23
Are you enthusiastic about life?	CADS	0.23
Do you like things that are exciting and loud?	CADS	0.22
I've been feeling loved	WEMWBS	0.22
You lie easily and skilfully	APSD	0.21
I hated myself	MFQ	-0.32
I had bad dreams	RCMAS	-0.32
I thought my family would be better off without me	MFQ	-0.33
I worried a lot of the time	RCMAS	-0.33
Someone molested me	CTQ	-0.33
I believe that I was sexually abused	CTQ	-0.34
Someone tried to make me do sexual things or watch sexual things.	CTQ	-0.34
Have you ever been drunk?	DASI	-0.34
I cried a lot	MFQ	-0.34
In the last month, how have you tried to hurt yourself without trying to kill yourself?	DASI	-0.35
I felt miserable or unhappy	MFQ	-0.35
Someone tried to touch me in a sexual way, or tried to make me touch them	CTQ	-0.35
During the last month, how often did you drink spirits?	DASI	-0.35
I thought there was nothing good for me in the future	MFQ	-0.36
During the last 30 days, about how often did you feel so sad that nothing could cheer you up?	K10	-0.36
I thought about dying	MFQ	-0.37
During the last 30 days, about how often did you feel depressed?	K10	-0.37
Age	Demographics	-0.38
I thought that life was not worth living	MFQ	-0.46
I thought about killing myself	MFQ	-0.47

Table S4: CCA connectivity correlations computed by correlating the brain connectivity variables and the brain canonical variates of the second CCA mode, computed using the permutation framework. Top 20 most positively and top 20 most negatively correlated brain

connections shown only. **Functional networks:** Default Mode Network (DMN); Dorsal Attention Network (DAN); Frontoparietal Network (FPT); Limbic Network (LMB); Somatomotor Network (SMT); Ventral Attention Network (VAN); Subcortex (SBC); Visual Network (VIS). **Anatomical regions:** [1]Anterior Cingulate and Medial Prefrontal Cortex; [2]Auditory Association Cortex; [3]Basal Ganglia; [4]Dorsal Stream Visual Cortex; [5]DorsoLateral Prefrontal Cortex; [6]Early Auditory Cortex; [7]Early Visual Cortex; [8]Hippocampus; [9]Inferior Frontal Cortex; [10]Inferior Parietal Cortex; [11]Insular and Frontal Opercular Cortex; [12]Lateral Temporal Cortex; [13]MT+ Complex and Neighboring Visual Areas; [14]Medial Temporal Cortex; [15]Orbital and Polar Frontal Cortex; [16]Paracentral Lobular and Mid Cingulate Cortex; [17]Posterior Cingulate Cortex; [18]Posterior Opercular Cortex; [19]Premotor Cortex; [20]Primary Visual Cortex; [21]Somatosensory and Motor Cortex; [22]Superior Parietal Cortex; [23]Temporo-Parieto-Occipital Junction; [24]Thalamus; [25]Ventral Stream Visual Cortex.

Label node A	Label node B	Correlation
Left-Area-8Ad (DMN)[5]	Left-Thalamus-Proper (SBC)[24]	0.38
Right-Area-dorsal-23-a+b (DMN)[17]	Left-Superior-Frontal-Language-Area (DMN)[5]	0.37
Right-Area-7m (DMN)[17]	Left-Thalamus-Proper (SBC)[24]	0.36
Left-Area-dorsal-23-a+b (DMN)[17]	Left-Superior-Frontal-Language-Area (DMN)[5]	0.35
Left-Area-posterior-24 (DMN)[1]	Left-Area-8Ad (DMN)[5]	0.35
Left-Area-PGs (DMN)[10]	Left-Thalamus-Proper (SBC)[24]	0.35
Left-Area-7m (DMN)[17]	Left-Thalamus-Proper (SBC)[24]	0.35
Right-Area-8Ad (DMN)[5]	Left-Thalamus-Proper (SBC)[24]	0.34
Right-Area-31p-ventral (DMN)[17]	Left-Superior-Frontal-Language-Area (DMN)[5]	0.34
Left-Area-posterior-24 (DMN)[1]	Left-Area-dorsal-23-a+b (DMN)[17]	0.34
Left-Area-posterior-24 (DMN)[1]	Left-Area-8Av (DMN)[5]	0.34
Right-Area-dorsal-23-a+b (DMN)[17]	Left-Area-posterior-24 (DMN)[1]	0.34
Left-Area-posterior-24 (DMN)[1]	Left-Area-PGs (DMN)[10]	0.33
Right-Area-5m (SMT)[16]	Right-Area-dorsal-23-a+b (DMN)[17]	0.32
Right-Area-8Ad (DMN)[5]	Left-Area-posterior-24 (DMN)[1]	0.32
Left-Area-TE1-posterior (DMN)[12]	Left-Area-9-Middle (DMN)[1]	0.32
Left-Superior-Frontal-Language-Area (DMN)[5]	Left-Thalamus-Proper (SBC)[24]	0.32
Left-Area-9-Middle (DMN)[1]	Left-Thalamus-Proper (SBC)[24]	0.32
Left-Area-TE1-posterior (DMN)[12]	Left-Area-9-anterior (DMN)[5]	0.32
Right-Area-dorsal-23-a+b (DMN)[17]	Left-Area-6m-anterior (VAN)[16]	0.32
Right-Area-PF-opercular (VAN)[10]	Right-Area-5m (SMT)[16]	-0.27
Right-Area-PFt (DAN)[10]	Left-Middle-Temporal-Area (VIS)[13]	-0.27
Right-Area-PFt (DAN)[10]	Left-Area-Lateral-Occipital-1 (VIS)[13]	-0.28
Right-RetroInsular-Cortex (SMT)[6]	Left-Area-PF-opercular (VAN)[10]	-0.28
Right-Area-PFt (DAN)[10]	Left-Seventh-Visual-Area (VIS)[4]	-0.28
Right-Area-PFcm (SMT)[6]	Left-Area-6m-anterior (VAN)[16]	-0.28

Right-ParaBelt-Complex (SMT)[6]	Left-Frontal-Opercular-Area-2 (SMT)[11]	-0.28
Right-Area-PFt (DAN)[10]	Left-Area-V3CD (VIS)[13]	-0.28
Right-Auditory-4-Complex (SMT)[2]	Left-Frontal-Opercular-Area-2 (SMT)[11]	-0.28
Right-Frontal-Opercular-Area-4 (VAN)[11]	Left-Area-Posterior-Insular-1 (VAN)[11]	-0.28
Right-Area-PFt (DAN)[10]	Right-Middle-Temporal-Area (VIS)[13]	-0.28
Right-Area-PFcm (SMT)[6]	Left-Area-Frontal-Opercular-5 (VAN)[11]	-0.29
Right-Area-V4t (VIS)[13]	Right-Area-PFt (DAN)[10]	-0.29
Right-Area-Lateral-Occipital-3 (VIS)[13]	Right-Area-PFt (DAN)[10]	-0.29
Right-Frontal-Opercular-Area-4 (VAN)[11]	Right-Area-PFcm (SMT)[6]	-0.30
Right-RetroInsular-Cortex (SMT)[6]	Left-Frontal-Opercular-Area-2 (SMT)[11]	-0.30
Right-Area-PF-opercular (VAN)[10]	Left-Area-6m-anterior (VAN)[16]	-0.30
Right-Area-PFt (DAN)[10]	Right-Area-Lateral-Occipital-2 (VIS)[13]	-0.31
Right-Area-5m-ventral (VAN)[16]	Left-Area-Frontal-Opercular-5 (VAN)[11]	-0.31
Right-Area-PFt (DAN)[10]	Right-Area-Lateral-Occipital-1 (VIS)[13]	-0.31

Table S5: CCA connectivity correlations computed by correlating the behavioural variables and the behavioural canonical variates of the first CCA mode, when using the multiple hold-out framework. Top 20 most positively and top 20 most negatively correlated items shown only.

Clinical label	Questionnaire	Correlation
Age	Demographics	0.53
During the last month, how often did you drink spirits?	DASI	0.48
In the last 6 months, how often have you been drunk in the way described in Q7?	DASI	0.47
Have you ever been drunk?	DASI	0.47
Male	Demographics	0.42
During the last month, how often did you drink beer or cider?	DASI	0.41
During the last month, how often did you smoke a cigarette/s?	DASI	0.37
During the last month, on the days you smoked, on average how many cigarettes did you smoke per day?	DASI	0.36
You engage in illegal activities	APSD	0.35
Do you react with little or no emotion to both positive and negative things?	CADS	0.32
Do you like TV, movies, comics, or electronic games with a lot of violence in them?	CADS	0.31
Do you enjoy doing things that are risky or dangerous?	CADS	0.31
I like to think about complex problems	BIS	0.29
I seem very cold and uncaring to others	ICU	0.28
You do risky or dangerous things	APSD	0.27
I do not show my emotions to others	ICU	0.26
During the last month, how often did you drink wine?	DASI	0.25
During the last month, how often did you take/use cannabis?	DASI	0.25

Are you brave?	CADS	0.24
Are you daring and adventurous?	CADS	0.23
I sometimes jump quickly from one topic to another when speaking	SPQ	-0.20
I worried what other people thought about me	RCMAS	-0.20
Do you want everyone to follow the rules, including yourself?	CADS	-0.20
I had the best family in the world	CTQ	-0.20
I often ramble on too much when speaking	SPQ	-0.21
I thought my family would be better off without me	MFQ	-0.21
I am concerned about the feelings of others	ICU	-0.21
Do you sometimes feel that other people are watching you?	SPQ	-0.22
Do you sometimes feel that people are talking about you?	SPQ	-0.22
When you see other people talking to each other, do you often wonder if they are talking about you?	SPQ	-0.22
My feelings got hurt easily when I was fussed at	RCMAS	-0.23
Do you like things to stay the same and not change?	CADS	-0.24
My feelings got hurt easily	RCMAS	-0.24
Do you get upset easily?	CADS	-0.24
I thought I looked ugly	MFQ	-0.25
Are you emotional?	CADS	-0.27
Are you easily embarrassed?	CADS	-0.29
I worried about what my parents would say to me	RCMAS	-0.32
Would you feel guilty if you did something that broke the law?	CADS	-0.36
Female	Demographics	-0.42

Table S6: CCA connectivity correlations computed by correlating the brain connectivity variables and the brain canonical variates of the first CCA mode, when using the multiple hold-out framework. Top 20 most positively and top 20 most negatively correlated brain connections shown only. **Functional networks:** Default Mode Network (DMN); Dorsal Attention Network (DAN); Frontoparietal Network (FPT); Limbic Network (LMB); Somatomotor Network (SMT); Ventral Attention Network (VAN); Subcortex (SBC); Visual Network (VIS). **Anatomical regions:** [1]Anterior Cingulate and Medial Prefrontal Cortex; [2]Auditory Association Cortex; [3]Basal Ganglia; [4]Dorsal Stream Visual Cortex; [5]DorsoLateral Prefrontal Cortex; [6]Early Auditory Cortex; [7]Early Visual Cortex; [8]Hippocampus; [9]Inferior Frontal Cortex; [10]Inferior Parietal Cortex; [11]Insular and Frontal Opercular Cortex; [12]Lateral Temporal Cortex; [13]MT+ Complex and Neighboring Visual Areas; [14]Medial Temporal Cortex; [15]Orbital and Polar Frontal Cortex; [16]Paracentral Lobular and Mid Cingulate Cortex; [17]Posterior Cingulate Cortex; [18]Posterior Opercular Cortex; [19]Premotor Cortex; [20]Primary Visual Cortex; [21]Somatosensory and Motor Cortex; [22]Superior Parietal Cortex; [23]Temporo-Parieto-Occipital Junction; [24]Thalamus; [25]Ventral Stream Visual Cortex.

**Label node A**	**Label node B**	**Correlation**
Right-Insular-Granular-Complex (SMT)[11]	Right-Third-Visual-Area (VIS)[7]	0.71

Right-Medial-Area-7A (DAN)[22]	Left-Insular-Granular-Complex (SMT)[11]	0.71
Right-Posterior-Insular-Area-2 (VAN)[11]	Left-Area-5m (SMT)[16]	0.71
Left-Insular-Granular-Complex (SMT)[11]	Left-Sixth-Visual-Area (VIS)[4]	0.71
Left-Insular-Granular-Complex (SMT)[11]	Left-Area-5m (SMT)[16]	0.71
Right-PreCuneus-Visual-Area (DAN)[17]	Left-Insular-Granular-Complex (SMT)[11]	0.71
Right-PreCuneus-Visual-Area (DAN)[17]	Left-Area-OP2-3/VS (SMT)[18]	0.71
Right-Insular-Granular-Complex (SMT)[11]	Left-Area-5m (SMT)[16]	0.70
Right-Area-OP2-3/VS (SMT)[18]	Left-Area-5m (SMT)[16]	0.70
Right-Insular-Granular-Complex (SMT)[11]	Right-Second-Visual-Area (VIS)[7]	0.70
Right-Area-23c (VAN)[16]	Left-Area-OP2-3/VS (SMT)[18]	0.70
Left-Area-OP2-3/VS (SMT)[18]	Left-Area-5m (SMT)[16]	0.70
Right-Insular-Granular-Complex (SMT)[11]	Left-Third-Visual-Area (VIS)[7]	0.70
Left-Insular-Granular-Complex (SMT)[11]	Left-PreCuneus-Visual-Area (DMN)[17]	0.70
Right-Insular-Granular-Complex (SMT)[11]	Left-Fourth-Visual-Area (VIS)[7]	0.70
Right-Second-Visual-Area (VIS)[7]	Left-Insular-Granular-Complex (SMT)[11]	0.70
Right-Medial-Area-7A (DAN)[22]	Left-Area-OP2-3/VS (SMT)[18]	0.69
Left-Area-OP2-3/VS (SMT)[18]	Left-Sixth-Visual-Area (VIS)[4]	0.69
Right-Area-V3B (VIS)[4]	Left-Insular-Granular-Complex (SMT)[11]	0.69
Right-Area-5m-ventral (VAN)[16]	Left-Area-OP2-3/VS (SMT)[18]	0.69
Right-Area-23d (DMN)[17]	Left-Area-31pd (DMN)[17]	-0.13
Left-Polar-10p (LMB)[15]	Left-Area-10v (LMB)[1]	-0.13
Right-Area-10v (LMB)[1]	Left-Parieto-Occipital-Sulcus-Area-2 (DMN)[17]	-0.13
Left-Area-posterior-10p (DMN)[15]	Left-Polar-10p (LMB)[15]	-0.13
Left-Area-ventral-23-a+b (DMN)[17]	Left-Area-23d (DMN)[17]	-0.13
Right-Area-10v (LMB)[1]	Left-Area-23d (DMN)[17]	-0.13
Left-Area-PGs (DMN)[10]	Left-Polar-10p (LMB)[15]	-0.13
Right-Area-23d (DMN)[17]	Left-Area-31p-ventral (DMN)[17]	-0.13
Left-Area-10v (LMB)[1]	Left-Area-23d (DMN)[17]	-0.14
Right-RetroSplenial-Complex (DMN)[17]	Left-Area-31pd (DMN)[17]	-0.14
Right-Area-10v (LMB)[1]	Left-Polar-10p (LMB)[15]	-0.14
Left-Area-31p-ventral (DMN)[17]	Left-Parieto-Occipital-Sulcus-Area-2 (DMN)[17]	-0.14
Left-Area-10v (LMB)[1]	Left-Parieto-Occipital-Sulcus-Area-2 (DMN)[17]	-0.14
Right-Area-8Ad (DMN)[5]	Left-Area-PGs (DMN)[10]	-0.15
Left-Area-dorsal-23-a+b (DMN)[17]	Left-Area-23d (DMN)[17]	-0.15
Right-Area-10v (LMB)[1]	Left-Area-p32 (DMN)[1]	-0.15
Left-Area-31p-ventral (DMN)[17]	Left-Area-23d (DMN)[17]	-0.15
Left-Area-31pd (DMN)[17]	Left-Area-23d (DMN)[17]	-0.16
Left-Area-31pd (DMN)[17]	Left-Parieto-Occipital-Sulcus-Area-2 (DMN)[17]	-0.17
Right-Polar-10p (LMB)[15]	Left-Polar-10p (LMB)[15]	-0.17

Table S7: Atlas with region MNI coordinates (x, y, z), region labels, anatomical and functional assignments.

X	Y	Z	Label	Anatomical region	Functional network
-12	-20	5	Left-Thalamus-Proper	Thalamus	Subcortical Network
-15	9	7	Left-Caudate	Basal Ganglia	Subcortical Network
-26	0	-2	Left-Putamen	Basal Ganglia	Subcortical Network
-26	-24	-15	Left-Hippocampus	Hippocampus	Subcortical Network
-24	-7	-21	Left-Amygdala	Basal Ganglia	Subcortical Network
-10	11	-9	Left-Accumbens-area	Basal Ganglia	Subcortical Network
12	-19	6	Right-Thalamus-Proper	Thalamus	Subcortical Network
16	9	8	Right-Caudate	Basal Ganglia	Subcortical Network
27	1	-2	Right-Putamen	Basal Ganglia	Subcortical Network
28	-23	-15	Right-Hippocampus	Hippocampus	Subcortical Network
25	-6	-21	Right-Amygdala	Basal Ganglia	Subcortical Network
10	10	-9	Right-Accumbens-area	Basal Ganglia	Subcortical Network
-12	-83	1	Left-Primary-Visual-Cortex	Primary Visual Cortex	Visual Network
-42	-67	4	Left-Medial-Superior-Temporal-Area	MT+ Complex and Neighboring Visual Areas	Dorsal Attention Network
-14	-79	28	Left-Sixth-Visual-Area	Dorsal Stream Visual Cortex	Visual Network
-12	-80	2	Left-Second-Visual-Area	Early Visual Cortex	Visual Network
-18	-85	5	Left-Third-Visual-Area	Early Visual Cortex	Visual Network
-30	-83	-4	Left-Fourth-Visual-Area	Early Visual Cortex	Visual Network
-33	-71	-15	Left-Eighth-Visual-Area	Ventral Stream Visual Cortex	Visual Network
-28	-20	54	Left-Primary-Motor-Cortex	Somatosensory and Motor Cortex	Somatomotor Network
-38	-23	51	Left-Primary-Sensory-Cortex	Somatosensory and Motor Cortex	Somatomotor Network
-40	-5	48	Left-Frontal-Eye-Fields	Premotor Cortex	Dorsal Attention Network
-48	-1	38	Left-Premotor-Eye-Field	Premotor Cortex	Dorsal Attention Network
-48	-1	46	Left-Area-55b	Premotor Cortex	Somatomotor Network
-16	-89	24	Left-Area-V3A	Dorsal Stream Visual Cortex	Visual Network
-6	-40	17	Left-RetroSplenial-Complex	Posterior Cingulate Cortex	Default Mode Network
-10	-70	34	Left-Parieto-Occipital-Sulcus-Area-2	Posterior Cingulate Cortex	Default Mode Network

-24	-82	26	Left-Seventh-Visual-Area	Dorsal Stream Visual Cortex	Visual Network
-23	-73	33	Left-IntraParietal-Sulcus-Area-1	Dorsal Stream Visual Cortex	Dorsal Attention Network
-41	-56	-20	Left-Fusiform-Face-Complex	Ventral Stream Visual Cortex	Visual Network
-27	-80	16	Left-Area-V3B	Dorsal Stream Visual Cortex	Visual Network
-39	-84	2	Left-Area-Lateral-Occipital-1	MT+ Complex and Neighboring Visual Areas	Visual Network
-43	-82	-7	Left-Area-Lateral-Occipital-2	MT+ Complex and Neighboring Visual Areas	Visual Network
-41	-80	-14	Left-Posterior-InferoTemporal-Complex	Ventral Stream Visual Cortex	Visual Network
-45	-73	6	Left-Middle-Temporal-Area	MT+ Complex and Neighboring Visual Areas	Visual Network
-44	-24	9	Left-Primary-Auditory-Cortex	Early Auditory Cortex	Somatomotor Network
-57	-45	21	Left-PeriSylvian-Language-Area	Temporo-Parieto-Occipital Junction	Ventral Attention Network
-8	17	61	Left-Superior-Frontal-Language-Area	DorsoLateral Prefrontal Cortex	Default Mode Network
-7	-50	48	Left-PreCuneus-Visual-Area	Posterior Cingulate Cortex	Default Mode Network
-60	-47	14	Left-Superior-Temporal-Visual-Area	Temporo-Parieto-Occipital Junction	Default Mode Network
-7	-66	48	Left-Medial-Area-7P	Superior Parietal Cortex	Frontoparietal Network
-5	-62	33	Left-Area-7m	Posterior Cingulate Cortex	Default Mode Network
-11	-59	13	Left-Parieto-Occipital-Sulcus-Area-1	Posterior Cingulate Cortex	Default Mode Network
-3	-20	37	Left-Area-23d	Posterior Cingulate Cortex	Default Mode Network
-5	-56	18	Left-Area-ventral-23-a+b	Posterior Cingulate Cortex	Default Mode Network
-3	-40	30	Left-Area-dorsal-23-a+b	Posterior Cingulate Cortex	Default Mode Network
-8	-45	32	Left-Area-31p-ventral	Posterior Cingulate Cortex	Default Mode Network
-7	-39	60	Left-Area-5m	Paracentral Lobular and Mid Cingulate Cortex	Somatomotor Network
-15	-36	48	Left-Area-5m-ventral	Paracentral Lobular and Mid Cingulate Cortex	Ventral Attention Network
-11	-28	42	Left-Area-23c	Paracentral Lobular and Mid Cingulate Cortex	Ventral Attention Network
-13	-46	69	Left-Area-5L	Paracentral Lobular and Mid Cingulate Cortex	Somatomotor Network
-8	-18	49	Left-Dorsal-Area-24d	Paracentral Lobular and Mid Cingulate Cortex	Somatomotor Network
-10	-2	43	Left-Ventral-Area-24d	Paracentral Lobular and Mid Cingulate Cortex	Somatomotor Network
-19	-53	64	Left-Lateral-Area-7A	Superior Parietal Cortex	Dorsal Attention Network
-7	3	56	Left-Supplementary-and-Cingulate-Eye-Field	Paracentral Lobular and Mid Cingulate Cortex	Ventral Attention Network
-17	4	66	Left-Area-6m-anterior	Paracentral Lobular and Mid Cingulate Cortex	Ventral Attention Network

-9	-58	59	Left-Medial-Area-7A	Superior Parietal Cortex	Dorsal Attention Network
-12	-72	51	Left-Lateral-Area-7P	Superior Parietal Cortex	Dorsal Attention Network
-34	-50	59	Left-Area-7PC	Superior Parietal Cortex	Dorsal Attention Network
-29	-56	53	Left-Area-Lateral-IntraParietal-ventral	Superior Parietal Cortex	Dorsal Attention Network
-21	-64	60	Left-Ventral-IntraParietal-Complex	Superior Parietal Cortex	Dorsal Attention Network
-22	-64	42	Left-Medial-IntraParietal-Area	Superior Parietal Cortex	Dorsal Attention Network
-44	-26	54	Left-Area-1	Somatosensory and Motor Cortex	Somatomotor Network
-37	-34	51	Left-Area-2	Somatosensory and Motor Cortex	Somatomotor Network
-34	-21	41	Left-Area-3a	Somatosensory and Motor Cortex	Somatomotor Network
-34	-14	64	Left-Dorsal-area-6	Premotor Cortex	Somatomotor Network
-12	-15	67	Left-Area-6mp	Paracentral Lobular and Mid Cingulate Cortex	Somatomotor Network
-58	2	30	Left-Ventral-Area-6	Premotor Cortex	Somatomotor Network
-4	-1	38	Left-Area-Posterior-24-prime	Anterior Cingulate and Medial Prefrontal Cortex	Ventral Attention Network
-4	12	28	Left-Area-33-prime	Anterior Cingulate and Medial Prefrontal Cortex	Frontoparietal Network
-5	19	29	Left-Anterior-24-prime	Anterior Cingulate and Medial Prefrontal Cortex	Ventral Attention Network
-10	15	37	Left-Area-p32-prime	Anterior Cingulate and Medial Prefrontal Cortex	Ventral Attention Network
-5	39	-1	Left-Area-a24	Anterior Cingulate and Medial Prefrontal Cortex	Default Mode Network
-9	39	23	Left-Area-dorsal-32	Anterior Cingulate and Medial Prefrontal Cortex	Default Mode Network
-6	31	43	Left-Area-8BM	Anterior Cingulate and Medial Prefrontal Cortex	Frontoparietal Network
-11	46	-1	Left-Area-p32	Anterior Cingulate and Medial Prefrontal Cortex	Default Mode Network
-7	50	-8	Left-Area-10r	Anterior Cingulate and Medial Prefrontal Cortex	Default Mode Network
-35	31	-15	Left-Area-47m	Orbital and Polar Frontal Cortex	Default Mode Network
-38	17	49	Left-Area-8Av	DorsoLateral Prefrontal Cortex	Default Mode Network
-23	26	42	Left-Area-8Ad	DorsoLateral Prefrontal Cortex	Default Mode Network
-7	52	24	Left-Area-9-Middle	Anterior Cingulate and Medial Prefrontal Cortex	Default Mode Network
-12	36	51	Left-Area-8B-Lateral	DorsoLateral Prefrontal Cortex	Default Mode Network
-17	45	38	Left-Area-9-Posterior	DorsoLateral Prefrontal Cortex	Default Mode Network
-11	63	9	Left-Area-10d	Orbital and Polar Frontal Cortex	Default Mode Network
-41	17	36	Left-Area-8C	DorsoLateral Prefrontal Cortex	Frontoparietal Network

-51	15	12	Left-Area-44	Inferior Frontal Cortex	Default Mode Network
-48	26	4	Left-Area-45	Inferior Frontal Cortex	Default Mode Network
-44	31	-11	Left-Area-47l-(47-lateral)	Inferior Frontal Cortex	Default Mode Network
-39	47	-10	Left-Area-anterior-47r	Inferior Frontal Cortex	Default Mode Network
-52	6	17	Left-Rostral-Area-6	Premotor Cortex	Ventral Attention Network
-41	12	24	Left-Area-IFJa	Inferior Frontal Cortex	Frontoparietal Network
-39	3	28	Left-Area-IFJp	Inferior Frontal Cortex	Dorsal Attention Network
-47	21	20	Left-Area-IFSp	Inferior Frontal Cortex	Frontoparietal Network
-45	31	11	Left-Area-IFSa	Inferior Frontal Cortex	Frontoparietal Network
-43	29	28	Left-Area-posterior-9-46v	DorsoLateral Prefrontal Cortex	Frontoparietal Network
-37	37	28	Left-Area-46	DorsoLateral Prefrontal Cortex	Frontoparietal Network
-37	48	9	Left-Area-anterior-9-46v	DorsoLateral Prefrontal Cortex	Frontoparietal Network
-29	43	21	Left-Area-9-46d	DorsoLateral Prefrontal Cortex	Frontoparietal Network
-19	54	25	Left-Area-9-anterior	DorsoLateral Prefrontal Cortex	Default Mode Network
-4	51	-18	Left-Area-10v	Anterior Cingulate and Medial Prefrontal Cortex	Limbic Network
-25	56	-6	Left-Area-anterior-10p	Orbital and Polar Frontal Cortex	Default Mode Network
-12	58	-16	Left-Polar-10p	Orbital and Polar Frontal Cortex	Limbic Network
-24	46	-14	Left-Area-11l	Orbital and Polar Frontal Cortex	Frontoparietal Network
-23	26	-19	Left-Area-13l	Orbital and Polar Frontal Cortex	Limbic Network
-32	21	-18	Left-Area-47s	Orbital and Polar Frontal Cortex	Default Mode Network
-29	-55	41	Left-Area-Lateral-IntraParietal-dorsal	Superior Parietal Cortex	Dorsal Attention Network
-25	-4	53	Left-Area-6-anterior	Premotor Cortex	Dorsal Attention Network
-32	7	53	Left-Inferior-6-8-Transitional-Area	DorsoLateral Prefrontal Cortex	Frontoparietal Network
-22	22	55	Left-Superior-6-8-Transitional-Area	DorsoLateral Prefrontal Cortex	Default Mode Network
-56	-1	9	Left-Area-43	Posterior Opercular Cortex	Ventral Attention Network
-58	-13	14	Left-Area-OP4/PV	Posterior Opercular Cortex	Somatomotor Network
-47	-22	17	Left-Area-OP1/SII	Posterior Opercular Cortex	Somatomotor Network
-40	-16	17	Left-Area-OP2-3/VS	Posterior Opercular Cortex	Somatomotor Network
-39	-22	-2	Left-Area-52	Early Auditory Cortex	Somatomotor Network

-40	-35	17	Left-RetroInsular-Cortex	Early Auditory Cortex	Somatomotor Network
-51	-32	19	Left-Area-PFcm	Early Auditory Cortex	Ventral Attention Network
-39	-4	-2	Left-Posterior-Insular-Area-2	Insular and Frontal Opercular Cortex	Ventral Attention Network
-51	0	-8	Left-Area-TA2	Auditory Association Cortex	Somatomotor Network
-40	12	6	Left-Frontal-Opercular-Area-4	Insular and Frontal Opercular Cortex	Ventral Attention Network
-36	9	1	Left-Middle-Insular-Area	Insular and Frontal Opercular Cortex	Ventral Attention Network
-30	5	-18	Left-Pirform-Cortex	Insular and Frontal Opercular Cortex	Limbic Network
-31	23	-3	Left-Anterior-Ventral-Insular-Area	Insular and Frontal Opercular Cortex	Frontoparietal Network
-34	13	-12	Left-Anterior-Agranular-Insula-Complex	Insular and Frontal Opercular Cortex	Default Mode Network
-50	3	4	Left-Frontal-Opercular-Area-1	Posterior Opercular Cortex	Ventral Attention Network
-35	3	12	Left-Frontal-Opercular-Area-3	Insular and Frontal Opercular Cortex	Ventral Attention Network
-42	-4	13	Left-Frontal-Opercular-Area-2	Insular and Frontal Opercular Cortex	Somatomotor Network
-50	-30	36	Left-Area-PFt	Inferior Parietal Cortex	Dorsal Attention Network
-36	-42	41	Left-Anterior-IntraParietal-Area	Superior Parietal Cortex	Dorsal Attention Network
-17	-35	-11	Left-PreSubiculum	Medial Temporal Cortex	Default Mode Network
-21	-55	2	Left-ProStriate-Area	Posterior Cingulate Cortex	Visual Network
-51	9	-18	Left-Area-STGa	Auditory Association Cortex	Default Mode Network
-54	-25	5	Left-ParaBelt-Complex	Early Auditory Cortex	Somatomotor Network
-61	-15	-4	Left-Auditory-5-Complex	Auditory Association Cortex	Somatomotor Network
-22	-35	-17	Left-ParaHippocampal-Area-1	Medial Temporal Cortex	Default Mode Network
-32	-38	-18	Left-ParaHippocampal-Area-3	Medial Temporal Cortex	Visual Network
-54	-8	-13	Left-Area-STSd-anterior	Auditory Association Cortex	Default Mode Network
-53	-33	-2	Left-Area-STSd-posterior	Auditory Association Cortex	Default Mode Network
-55	-36	-7	Left-Area-STSv-posterior	Auditory Association Cortex	Default Mode Network
-60	-11	-23	Left-Area-TE1-anterior	Lateral Temporal Cortex	Default Mode Network
-60	-46	-13	Left-Area-TE1-posterior	Lateral Temporal Cortex	Default Mode Network
-58	-57	-1	Left-Area-PHT	Lateral Temporal Cortex	Dorsal Attention Network
-47	-60	-11	Left-Area-PH	MT+ Complex and Neighboring Visual Areas	Dorsal Attention Network
-55	-45	7	Left-Area-TemporoParietoOccipital-Junction-1	Temporo-Parieto-Occipital Junction	Somatomotor Network

-49	-61	10	Left-Area-TemporoParietoOccipital-Junction-2	Temporo-Parieto-Occipital Junction	Dorsal Attention Network
-44	-71	15	Left-Area-TemporoParietoOccipital-Junction-3	Temporo-Parieto-Occipital Junction	Dorsal Attention Network
-18	-71	28	Left-Dorsal-Transitional-Visual-Area	Posterior Cingulate Cortex	Visual Network
-40	-81	21	Left-Area-PGp	Inferior Parietal Cortex	Dorsal Attention Network
-40	-51	38	Left-Area-IntraParietal-2	Inferior Parietal Cortex	Frontoparietal Network
-31	-69	39	Left-Area-IntraParietal-1	Inferior Parietal Cortex	Frontoparietal Network
-32	-76	24	Left-Area-IntraParietal-0	Inferior Parietal Cortex	Dorsal Attention Network
-61	-24	25	Left-Area-PF-opercular	Inferior Parietal Cortex	Ventral Attention Network
-57	-39	36	Left-Area-PFm-Complex	Inferior Parietal Cortex	Ventral Attention Network
-48	-58	39	Left-Area-PFm-Complex	Inferior Parietal Cortex	Default Mode Network
-44	-59	22	Left-Area-PGi	Inferior Parietal Cortex	Default Mode Network
-40	-73	35	Left-Area-PGs	Inferior Parietal Cortex	Default Mode Network
-19	-85	37	Left-Area-V6A	Dorsal Stream Visual Cortex	Visual Network
-19	-53	-8	Left-VentroMedial-Visual-Area-1	Ventral Stream Visual Cortex	Visual Network
-29	-60	-11	Left-VentroMedial-Visual-Area-3	Ventral Stream Visual Cortex	Visual Network
-31	-36	-15	Left-ParaHippocampal-Area-2	Medial Temporal Cortex	Visual Network
-44	-77	-2	Left-Area-V4t	MT+ Complex and Neighboring Visual Areas	Visual Network
-47	-65	-2	Left-Area-FST	MT+ Complex and Neighboring Visual Areas	Dorsal Attention Network
-35	-86	11	Left-Area-V3CD	MT+ Complex and Neighboring Visual Areas	Visual Network
-46	-79	9	Left-Area-Lateral-Occipital-3	MT+ Complex and Neighboring Visual Areas	Visual Network
-28	-53	-7	Left-VentroMedial-Visual-Area-2	Ventral Stream Visual Cortex	Visual Network
-11	-52	34	Left-Area-31pd	Posterior Cingulate Cortex	Default Mode Network
-6	-37	42	Left-Area-31a	Posterior Cingulate Cortex	Default Mode Network
-31	-52	-19	Left-Ventral-Visual-Complex	Ventral Stream Visual Cortex	Visual Network
-5	23	-13	Left-Area-25	Anterior Cingulate and Medial Prefrontal Cortex	Limbic Network
-7	34	-14	Left-Area-s32	Anterior Cingulate and Medial Prefrontal Cortex	Limbic Network
-38	-13	-5	Left-Area-Posterior-Insular-1	Insular and Frontal Opercular Cortex	Ventral Attention Network
-36	-16	13	Left-Insular-Granular-Complex	Insular and Frontal Opercular Cortex	Somatomotor Network
-35	26	5	Left-Area-Frontal-Opercular-5	Insular and Frontal Opercular Cortex	Ventral Attention Network

-22	59	4	Left-Area-posterior-10p	Orbital and Polar Frontal Cortex	Default Mode Network
-43	40	0	Left-Area-posterior-47r	Inferior Frontal Cortex	Frontoparietal Network
-45	-18	2	Left-Medial-Belt-Complex	Early Auditory Cortex	Somatomotor Network
-46	-27	6	Left-Lateral-Belt-Complex	Early Auditory Cortex	Somatomotor Network
-63	-22	5	Left-Auditory-4-Complex	Auditory Association Cortex	Somatomotor Network
-52	-13	-18	Left-Area-STSv-anterior	Auditory Association Cortex	Default Mode Network
-44	-5	-16	Left-Para-Insular-Area	Insular and Frontal Opercular Cortex	Ventral Attention Network
-10	29	27	Left-Area-anterior-32-prime	Anterior Cingulate and Medial Prefrontal Cortex	Frontoparietal Network
-4	35	15	Left-Area-posterior-24	Anterior Cingulate and Medial Prefrontal Cortex	Default Mode Network
12	-81	2	Right-Primary-Visual-Cortex	Primary Visual Cortex	Visual Network
43	-66	3	Right-Medial-Superior-Temporal-Area	MT+ Complex and Neighboring Visual Areas	Visual Network
17	-77	29	Right-Sixth-Visual-Area	Dorsal Stream Visual Cortex	Visual Network
13	-79	3	Right-Second-Visual-Area	Early Visual Cortex	Visual Network
19	-85	6	Right-Third-Visual-Area	Early Visual Cortex	Visual Network
31	-84	-3	Right-Fourth-Visual-Area	Early Visual Cortex	Visual Network
31	-71	-13	Right-Eighth-Visual-Area	Ventral Stream Visual Cortex	Visual Network
29	-17	54	Right-Primary-Motor-Cortex	Somatosensory and Motor Cortex	Somatomotor Network
37	-22	51	Right-Primary-Sensory-Cortex	Somatosensory and Motor Cortex	Somatomotor Network
42	-3	49	Right-Frontal-Eye-Fields	Premotor Cortex	Dorsal Attention Network
45	2	36	Right-Premotor-Eye-Field	Premotor Cortex	Dorsal Attention Network
49	0	45	Right-Area-55b	Premotor Cortex	Ventral Attention Network
16	-87	29	Right-Area-V3A	Dorsal Stream Visual Cortex	Visual Network
6	-37	20	Right-RetroSplenial-Complex	Posterior Cingulate Cortex	Default Mode Network
11	-69	36	Right-Parieto-Occipital-Sulcus-Area-2	Posterior Cingulate Cortex	Frontoparietal Network
25	-81	30	Right-Seventh-Visual-Area	Dorsal Stream Visual Cortex	Visual Network
25	-69	35	Right-IntraParietal-Sulcus-Area-1	Dorsal Stream Visual Cortex	Dorsal Attention Network
39	-55	-19	Right-Fusiform-Face-Complex	Ventral Stream Visual Cortex	Visual Network
29	-75	20	Right-Area-V3B	Dorsal Stream Visual Cortex	Visual Network
39	-83	3	Right-Area-Lateral-Occipital-1	MT+ Complex and Neighboring Visual Areas	Visual Network

44	-82	-5	Right-Area-Lateral-Occipital-2	MT+ Complex and Neighboring Visual Areas	Visual Network
43	-79	-14	Right-Posterior-InferoTemporal-Complex	Ventral Stream Visual Cortex	Visual Network
47	-72	6	Right-Middle-Temporal-Area	MT+ Complex and Neighboring Visual Areas	Visual Network
44	-23	10	Right-Primary-Auditory-Cortex	Early Auditory Cortex	Somatomotor Network
61	-39	21	Right-PeriSylvian-Language-Area	Temporo-Parieto-Occipital Junction	Ventral Attention Network
9	16	63	Right-Superior-Frontal-Language-Area	DorsoLateral Prefrontal Cortex	Default Mode Network
7	-52	49	Right-PreCuneus-Visual-Area	Posterior Cingulate Cortex	Dorsal Attention Network
57	-42	14	Right-Superior-Temporal-Visual-Area	Temporo-Parieto-Occipital Junction	Ventral Attention Network
6	-66	49	Right-Medial-Area-7P	Superior Parietal Cortex	Frontoparietal Network
5	-60	33	Right-Area-7m	Posterior Cingulate Cortex	Default Mode Network
11	-58	15	Right-Parieto-Occipital-Sulcus-Area-1	Posterior Cingulate Cortex	Default Mode Network
4	-23	38	Right-Area-23d	Posterior Cingulate Cortex	Default Mode Network
5	-53	18	Right-Area-ventral-23-a+b	Posterior Cingulate Cortex	Default Mode Network
4	-41	31	Right-Area-dorsal-23-a+b	Posterior Cingulate Cortex	Default Mode Network
8	-45	33	Right-Area-31p-ventral	Posterior Cingulate Cortex	Default Mode Network
5	-38	63	Right-Area-5m	Paracentral Lobular and Mid Cingulate Cortex	Somatomotor Network
13	-38	51	Right-Area-5m-ventral	Paracentral Lobular and Mid Cingulate Cortex	Ventral Attention Network
11	-32	42	Right-Area-23c	Paracentral Lobular and Mid Cingulate Cortex	Ventral Attention Network
12	-44	71	Right-Area-5L	Paracentral Lobular and Mid Cingulate Cortex	Somatomotor Network
8	-18	50	Right-Dorsal-Area-24d	Paracentral Lobular and Mid Cingulate Cortex	Somatomotor Network
11	-5	46	Right-Ventral-Area-24d	Paracentral Lobular and Mid Cingulate Cortex	Somatomotor Network
20	-52	64	Right-Lateral-Area-7A	Superior Parietal Cortex	Dorsal Attention Network
7	4	57	Right-Supplementary-and-Cingulate-Eye-Field	Paracentral Lobular and Mid Cingulate Cortex	Ventral Attention Network
18	4	64	Right-Area-6m-anterior	Paracentral Lobular and Mid Cingulate Cortex	Ventral Attention Network
9	-60	60	Right-Medial-Area-7A	Superior Parietal Cortex	Dorsal Attention Network
13	-70	54	Right-Lateral-Area-7P	Superior Parietal Cortex	Dorsal Attention Network
33	-49	61	Right-Area-7PC	Superior Parietal Cortex	Dorsal Attention Network
28	-54	53	Right-Area-Lateral-IntraParietal-ventral	Superior Parietal Cortex	Dorsal Attention Network
19	-61	61	Right-Ventral-IntraParietal-Complex	Superior Parietal Cortex	Dorsal Attention Network

24	-64	45	Right-Medial-IntraParietal-Area	Superior Parietal Cortex	Dorsal Attention Network
45	-24	54	Right-Area-1	Somatosensory and Motor Cortex	Somatomotor Network
37	-32	51	Right-Area-2	Somatosensory and Motor Cortex	Somatomotor Network
31	-21	45	Right-Area-3a	Somatosensory and Motor Cortex	Somatomotor Network
36	-12	62	Right-Dorsal-area-6	Premotor Cortex	Somatomotor Network
15	-12	66	Right-Area-6mp	Paracentral Lobular and Mid Cingulate Cortex	Somatomotor Network
58	4	29	Right-Ventral-Area-6	Premotor Cortex	Somatomotor Network
5	-2	40	Right-Area-Posterior-24-prime	Anterior Cingulate and Medial Prefrontal Cortex	Ventral Attention Network
4	11	30	Right-Area-33-prime	Anterior Cingulate and Medial Prefrontal Cortex	Ventral Attention Network
5	18	32	Right-Anterior-24-prime	Anterior Cingulate and Medial Prefrontal Cortex	Ventral Attention Network
11	14	39	Right-Area-p32-prime	Anterior Cingulate and Medial Prefrontal Cortex	Ventral Attention Network
6	38	0	Right-Area-a24	Anterior Cingulate and Medial Prefrontal Cortex	Default Mode Network
10	37	24	Right-Area-dorsal-32	Anterior Cingulate and Medial Prefrontal Cortex	Default Mode Network
6	28	46	Right-Area-8BM	Anterior Cingulate and Medial Prefrontal Cortex	Frontoparietal Network
11	44	-2	Right-Area-p32	Anterior Cingulate and Medial Prefrontal Cortex	Default Mode Network
8	47	-8	Right-Area-10r	Anterior Cingulate and Medial Prefrontal Cortex	Default Mode Network
34	30	-16	Right-Area-47m	Orbital and Polar Frontal Cortex	Default Mode Network
38	20	47	Right-Area-8Av	DorsoLateral Prefrontal Cortex	Frontoparietal Network
23	27	44	Right-Area-8Ad	DorsoLateral Prefrontal Cortex	Default Mode Network
8	52	22	Right-Area-9-Middle	Anterior Cingulate and Medial Prefrontal Cortex	Default Mode Network
11	38	49	Right-Area-8B-Lateral	DorsoLateral Prefrontal Cortex	Default Mode Network
18	47	34	Right-Area-9-Posterior	DorsoLateral Prefrontal Cortex	Default Mode Network
11	64	6	Right-Area-10d	Orbital and Polar Frontal Cortex	Default Mode Network
39	18	35	Right-Area-8C	DorsoLateral Prefrontal Cortex	Frontoparietal Network
52	17	12	Right-Area-44	Inferior Frontal Cortex	Frontoparietal Network
50	27	3	Right-Area-45	Inferior Frontal Cortex	Default Mode Network
39	48	-7	Right-Area-anterior-47r	Inferior Frontal Cortex	Frontoparietal Network
50	8	16	Right-Rostral-Area-6	Premotor Cortex	Ventral Attention Network
41	18	22	Right-Area-IFJa	Inferior Frontal Cortex	Frontoparietal Network

37	8	27	Right-Area-IFJp	Inferior Frontal Cortex	Dorsal Attention Network
47	27	17	Right-Area-IFSp	Inferior Frontal Cortex	Frontoparietal Network
47	34	6	Right-Area-IFSa	Inferior Frontal Cortex	Frontoparietal Network
44	31	28	Right-Area-posterior-9-46v	DorsoLateral Prefrontal Cortex	Frontoparietal Network
36	37	27	Right-Area-46	DorsoLateral Prefrontal Cortex	Frontoparietal Network
39	49	11	Right-Area-anterior-9-46v	DorsoLateral Prefrontal Cortex	Frontoparietal Network
29	45	23	Right-Area-9-46d	DorsoLateral Prefrontal Cortex	Frontoparietal Network
18	58	21	Right-Area-9-anterior	DorsoLateral Prefrontal Cortex	Default Mode Network
5	49	-15	Right-Area-10v	Anterior Cingulate and Medial Prefrontal Cortex	Limbic Network
25	58	-7	Right-Area-anterior-10p	Orbital and Polar Frontal Cortex	Frontoparietal Network
13	58	-16	Right-Polar-10p	Orbital and Polar Frontal Cortex	Limbic Network
21	24	-20	Right-Area-13l	Orbital and Polar Frontal Cortex	Limbic Network
32	21	-18	Right-Area-47s	Orbital and Polar Frontal Cortex	Default Mode Network
30	-54	43	Right-Area-Lateral-IntraParietal-dorsal	Superior Parietal Cortex	Dorsal Attention Network
27	-2	51	Right-Area-6-anterior	Premotor Cortex	Dorsal Attention Network
35	11	54	Right-Inferior-6-8-Transitional-Area	DorsoLateral Prefrontal Cortex	Frontoparietal Network
20	19	56	Right-Superior-6-8-Transitional-Area	DorsoLateral Prefrontal Cortex	Frontoparietal Network
56	-2	10	Right-Area-43	Posterior Opercular Cortex	Somatomotor Network
58	-14	16	Right-Area-OP4/PV	Posterior Opercular Cortex	Somatomotor Network
44	-22	18	Right-Area-OP1/SII	Posterior Opercular Cortex	Somatomotor Network
40	-15	18	Right-Area-OP2-3/VS	Posterior Opercular Cortex	Somatomotor Network
39	-23	2	Right-Area-52	Early Auditory Cortex	Somatomotor Network
42	-33	17	Right-RetroInsular-Cortex	Early Auditory Cortex	Somatomotor Network
48	-30	22	Right-Area-PFcm	Early Auditory Cortex	Somatomotor Network
39	-5	-1	Right-Posterior-Insular-Area-2	Insular and Frontal Opercular Cortex	Ventral Attention Network
52	-1	-7	Right-Area-TA2	Auditory Association Cortex	Somatomotor Network
40	13	7	Right-Frontal-Opercular-Area-4	Insular and Frontal Opercular Cortex	Ventral Attention Network
38	8	1	Right-Middle-Insular-Area	Insular and Frontal Opercular Cortex	Ventral Attention Network
34	5	-18	Right-Pirform-Cortex	Insular and Frontal Opercular Cortex	Ventral Attention Network

34	23	-3	Right-Anterior-Ventral-Insular-Area	Insular and Frontal Opercular Cortex	Frontoparietal Network
35	13	-12	Right-Anterior-Agranular-Insula-Complex	Insular and Frontal Opercular Cortex	Ventral Attention Network
48	3	5	Right-Frontal-Opercular-Area-1	Posterior Opercular Cortex	Ventral Attention Network
35	6	11	Right-Frontal-Opercular-Area-3	Insular and Frontal Opercular Cortex	Ventral Attention Network
41	-4	15	Right-Frontal-Opercular-Area-2	Insular and Frontal Opercular Cortex	Somatomotor Network
52	-27	40	Right-Area-PFt	Inferior Parietal Cortex	Dorsal Attention Network
37	-41	42	Right-Anterior-IntraParietal-Area	Superior Parietal Cortex	Dorsal Attention Network
18	-35	-8	Right-PreSubiculum	Medial Temporal Cortex	Visual Network
20	-51	1	Right-ProStriate-Area	Posterior Cingulate Cortex	Visual Network
51	10	-19	Right-Area-STGa	Auditory Association Cortex	Default Mode Network
57	-18	5	Right-ParaBelt-Complex	Early Auditory Cortex	Somatomotor Network
61	-17	-3	Right-Auditory-5-Complex	Auditory Association Cortex	Somatomotor Network
23	-34	-17	Right-ParaHippocampal-Area-1	Medial Temporal Cortex	Visual Network
53	-7	-14	Right-Area-STSd-anterior	Auditory Association Cortex	Default Mode Network
49	-29	-3	Right-Area-STSd-posterior	Auditory Association Cortex	Default Mode Network
59	-33	-7	Right-Area-STSv-posterior	Auditory Association Cortex	Default Mode Network
60	-7	-25	Right-Area-TE1-anterior	Lateral Temporal Cortex	Default Mode Network
61	-44	-13	Right-Area-TE1-posterior	Lateral Temporal Cortex	Frontoparietal Network
59	-53	-4	Right-Area-PHT	Lateral Temporal Cortex	Dorsal Attention Network
49	-62	-11	Right-Area-PH	MT+ Complex and Neighboring Visual Areas	Dorsal Attention Network
54	-43	7	Right-Area-TemporoParietoOccipital-Junction-1	Temporo-Parieto-Occipital Junction	Ventral Attention Network
54	-58	7	Right-Area-TemporoParietoOccipital-Junction-2	Temporo-Parieto-Occipital Junction	Dorsal Attention Network
41	-64	14	Right-Area-TemporoParietoOccipital-Junction-3	Temporo-Parieto-Occipital Junction	Dorsal Attention Network
20	-67	28	Right-Dorsal-Transitional-Visual-Area	Posterior Cingulate Cortex	Visual Network
41	-79	23	Right-Area-PGp	Inferior Parietal Cortex	Dorsal Attention Network
43	-45	41	Right-Area-IntraParietal-2	Inferior Parietal Cortex	Frontoparietal Network
35	-66	42	Right-Area-IntraParietal-1	Inferior Parietal Cortex	Frontoparietal Network
33	-73	27	Right-Area-IntraParietal-0	Inferior Parietal Cortex	Dorsal Attention Network
60	-21	27	Right-Area-PF-opercular	Inferior Parietal Cortex	Ventral Attention Network

58	-34	36	Right-Area-PFm-Complex	Inferior Parietal Cortex	Ventral Attention Network
52	-50	39	Right-Area-PFm-Complex	Inferior Parietal Cortex	Frontoparietal Network
47	-59	23	Right-Area-PGi	Inferior Parietal Cortex	Default Mode Network
43	-67	38	Right-Area-PGs	Inferior Parietal Cortex	Default Mode Network
22	-81	41	Right-Area-V6A	Dorsal Stream Visual Cortex	Visual Network
19	-53	-8	Right-VentroMedial-Visual-Area-1	Ventral Stream Visual Cortex	Visual Network
29	-59	-10	Right-VentroMedial-Visual-Area-3	Ventral Stream Visual Cortex	Visual Network
32	-35	-15	Right-ParaHippocampal-Area-2	Medial Temporal Cortex	Visual Network
45	-78	-2	Right-Area-V4t	MT+ Complex and Neighboring Visual Areas	Visual Network
46	-64	-3	Right-Area-FST	MT+ Complex and Neighboring Visual Areas	Dorsal Attention Network
34	-83	14	Right-Area-V3CD	MT+ Complex and Neighboring Visual Areas	Visual Network
46	-77	9	Right-Area-Lateral-Occipital-3	MT+ Complex and Neighboring Visual Areas	Visual Network
29	-54	-7	Right-VentroMedial-Visual-Area-2	Ventral Stream Visual Cortex	Visual Network
12	-52	35	Right-Area-31pd	Posterior Cingulate Cortex	Default Mode Network
6	-42	41	Right-Area-31a	Posterior Cingulate Cortex	Frontoparietal Network
30	-48	-19	Right-Ventral-Visual-Complex	Ventral Stream Visual Cortex	Visual Network
5	20	-14	Right-Area-25	Anterior Cingulate and Medial Prefrontal Cortex	Limbic Network
6	33	-13	Right-Area-s32	Anterior Cingulate and Medial Prefrontal Cortex	Default Mode Network
39	-13	-4	Right-Area-Posterior-Insular-1	Insular and Frontal Opercular Cortex	Ventral Attention Network
36	-15	14	Right-Insular-Granular-Complex	Insular and Frontal Opercular Cortex	Somatomotor Network
37	26	5	Right-Area-Frontal-Opercular-5	Insular and Frontal Opercular Cortex	Ventral Attention Network
25	58	5	Right-Area-posterior-10p	Orbital and Polar Frontal Cortex	Frontoparietal Network
45	41	-3	Right-Area-posterior-47r	Inferior Frontal Cortex	Frontoparietal Network
45	-19	4	Right-Medial-Belt-Complex	Early Auditory Cortex	Somatomotor Network
49	-26	8	Right-Lateral-Belt-Complex	Early Auditory Cortex	Somatomotor Network
64	-17	4	Right-Auditory-4-Complex	Auditory Association Cortex	Somatomotor Network
56	-14	-17	Right-Area-STSv-anterior	Auditory Association Cortex	Default Mode Network
63	-27	-16	Right-Area-TE1-Middle	Lateral Temporal Cortex	Default Mode Network
45	-7	-13	Right-Para-Insular-Area	Insular and Frontal Opercular Cortex	Ventral Attention Network

10	28	28	Right-Area-anterior-32-prime	Anterior Cingulate and Medial Prefrontal Cortex	Frontoparietal Network
5	35	16	Right-Area-posterior-24	Anterior Cingulate and Medial Prefrontal Cortex	Default Mode Network

Figures

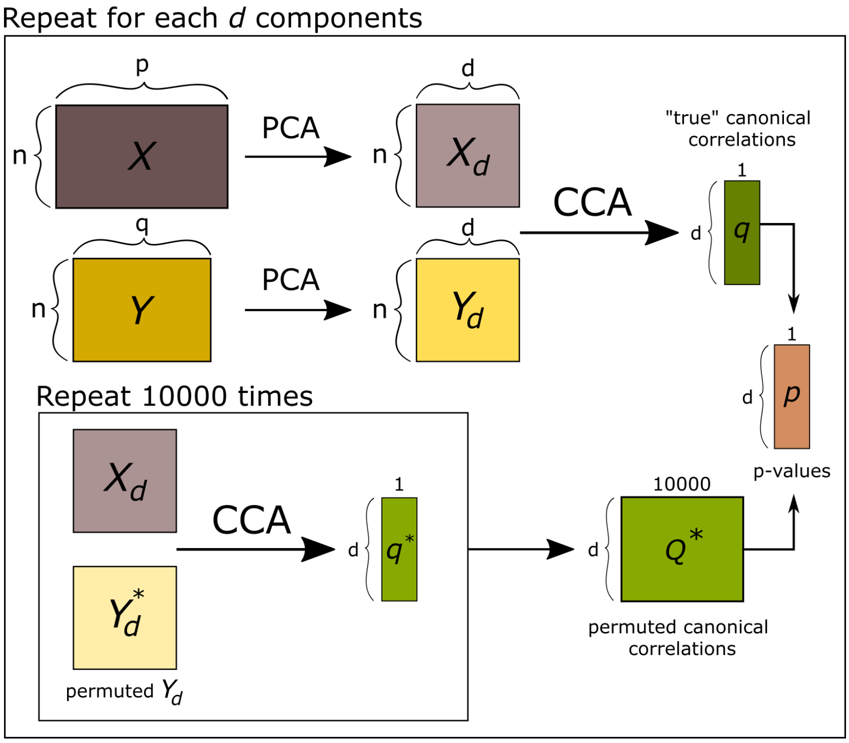

Figure S1: Permutation framework used to jointly find the optimal number of PCA components and estimate the statistically significant CCA modes.

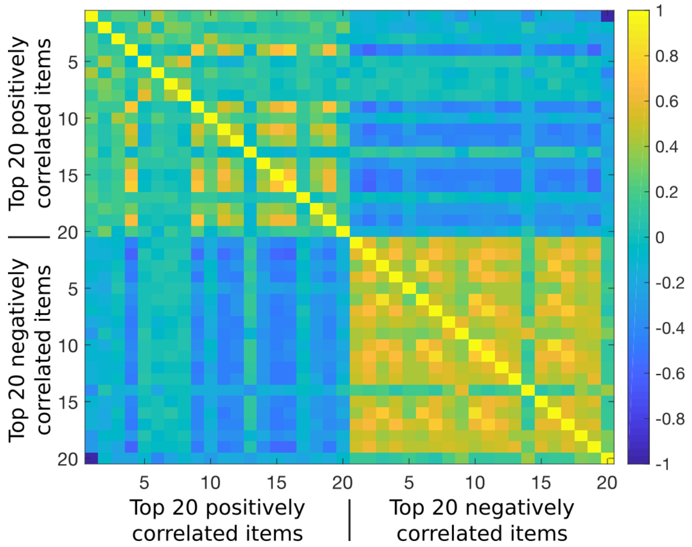

Figure S2: Correlations between the top 20 positive and top 20 negative behavioural items of the first CCA mode. Male sex (first item) is weakly associated with the other positive behavioural items (items 2-19 with mean correlation=0.20). Female sex (last item) is weakly associated with the other negative behavioural items (items 1-19 with mean correlation=0.17).

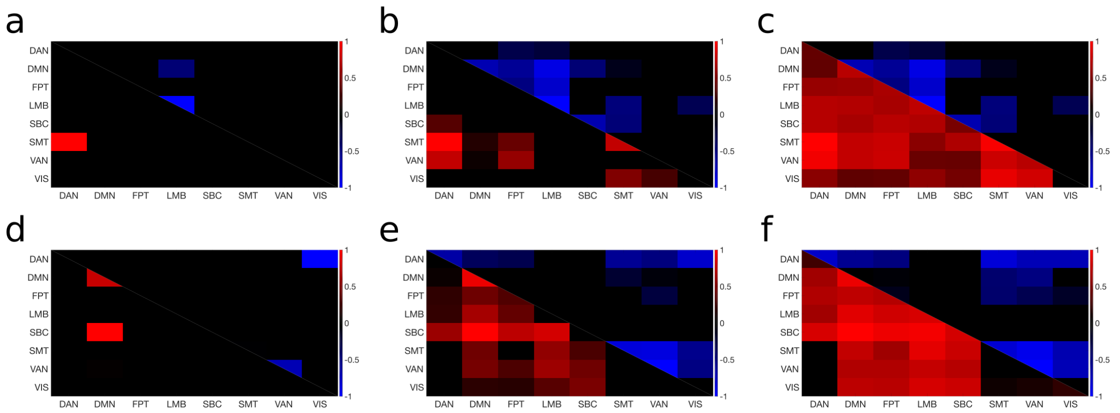

Figure S3: Mean correlations between and within resting-state networks for the first (**a-c**) and the second (**d-f**) CCA mode at three different levels of top connections: top 20 (**a, d**), top 0.5% (**b, e**) and top 5% (**c, f**) of most positively/negatively correlated connections. Positive correlations (red) and negative correlations (blue) are summarized separately in the lower and upper triangular matrices, respectively. The mean absolute correlations are log-transformed and normalized for easier comparison between the three levels. Dorsal Attention Network (DAN); Default Mode Network (DMN); Frontoparietal Network (FPT); Limbic Network (LMB); Subcortex (SBC); Somatomotor Network (SMT); Ventral Attention Network (VAN); Visual Network (VIS).

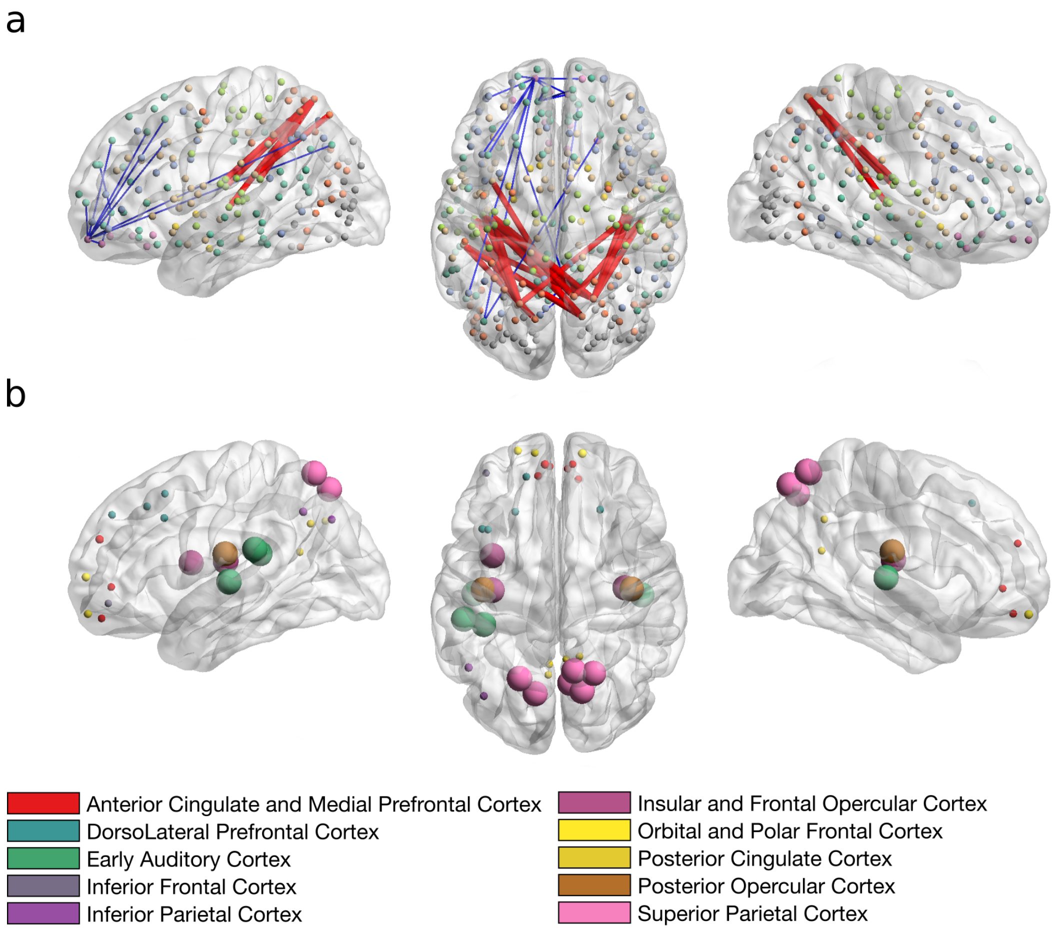

Figure S4: Correlations between the brain connectivity variables and the brain canonical variate (brain scores of all subjects) of the first CCA mode in sagittal (left and right) and axial views (middle). Notations are as in Fig. 4 of the main text except that nodes are colour coded by gross anatomical regions used in Glasser et al 2016[17]. The list of correlation values and respective labels can be found in Supplementary Table S2.

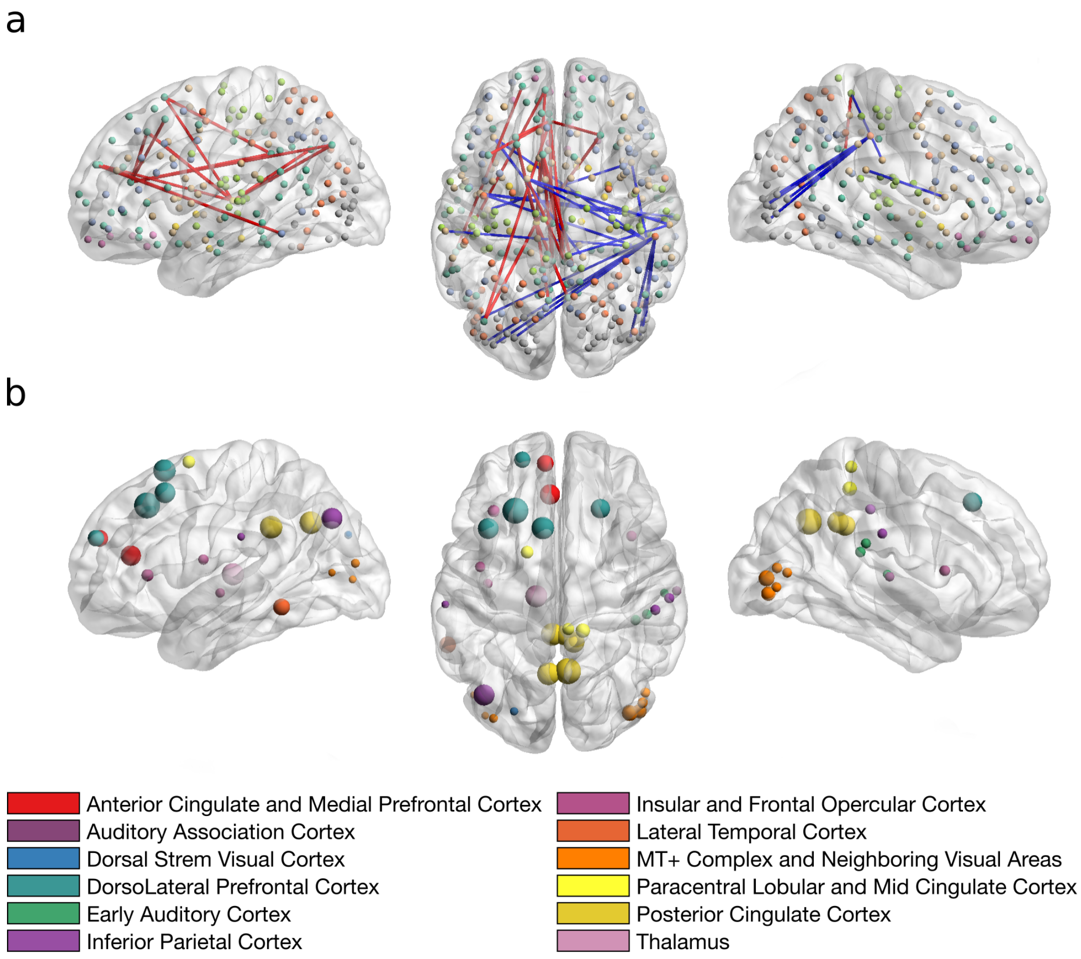

Figure S5: Correlations between the brain connectivity variables and the brain canonical variate (brain scores of all subjects) of the second CCA mode in sagittal (left and right) and axial views (middle). Notations are as in Fig. 5 of the main text except that nodes are colour coded by gross anatomical regions used in Glasser et al 2016[17]. The list of correlation values and respective labels can be found in Supplementary Table S4.

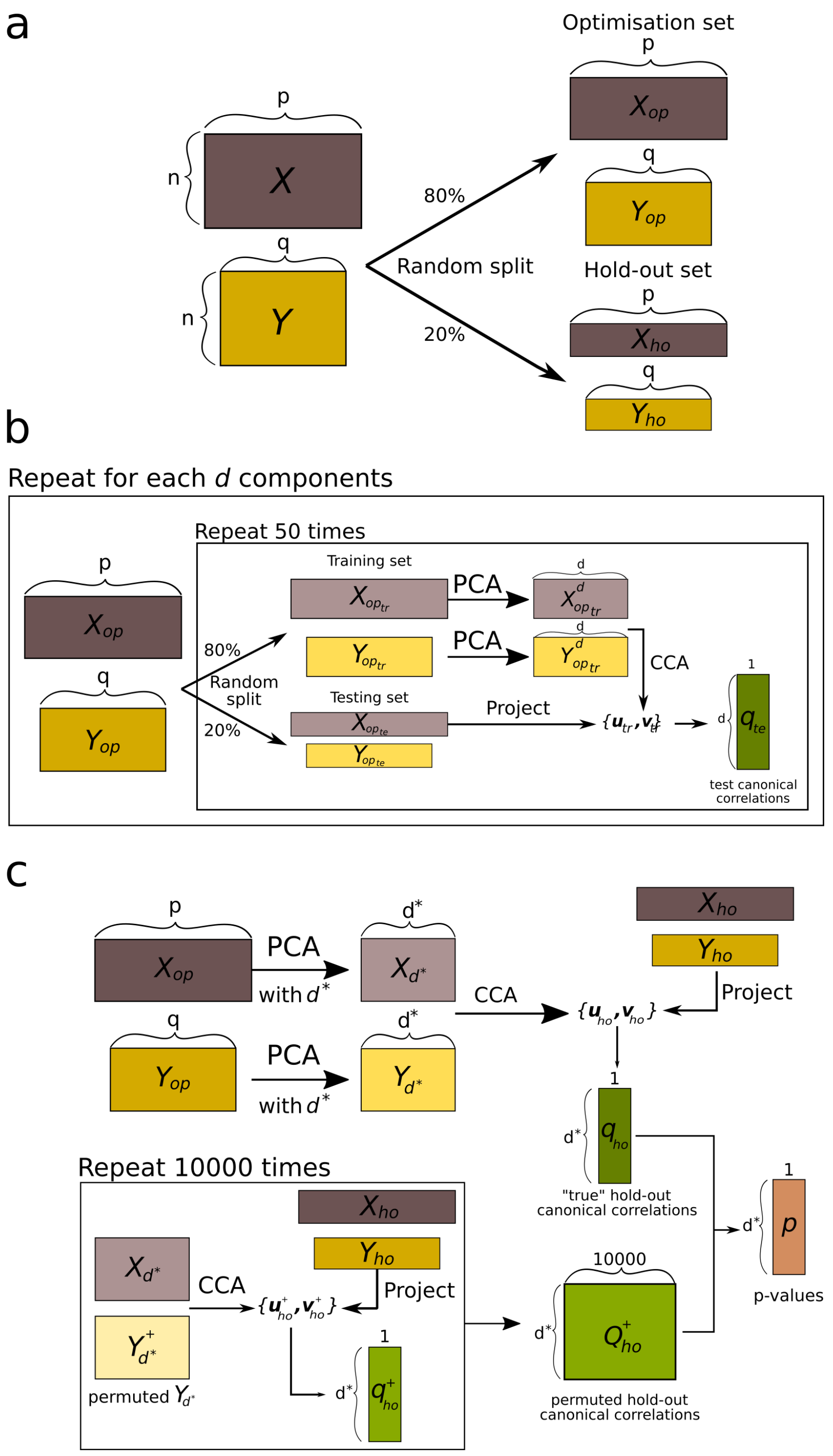

Figure S6: Hold-out framework used to jointly find the optimal number of PCA components and estimate the statistically significant CCA modes. **(a)** Random split of the data into optimisation and hold-out sets; **(b)** optimisation of the number of PCA components; **(c)** validation of the model using a hold-out set.

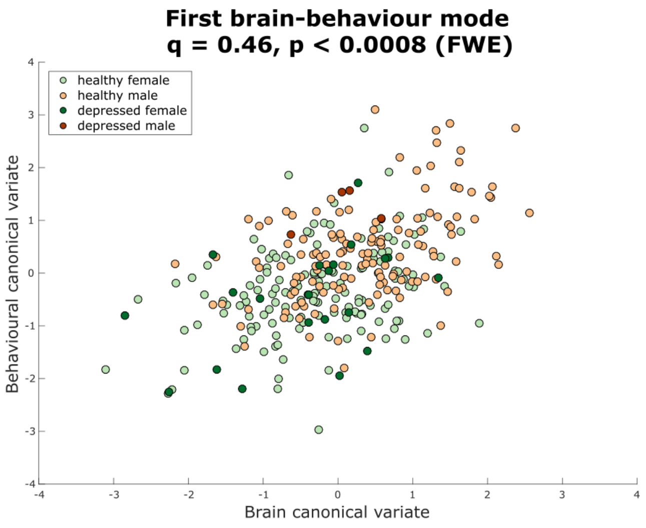

Figure S7: Significant brain-behaviour mode of covariation using the multiple hold-out framework. Scatter plot showing the brain and behaviour scores for the first CCA mode, where each dot represents an individual subject. Subjects are colour coded by sex and clinical diagnosis. The canonical hold-out correlation, *q*, and corresponding p-value are shown on the top of the plot.

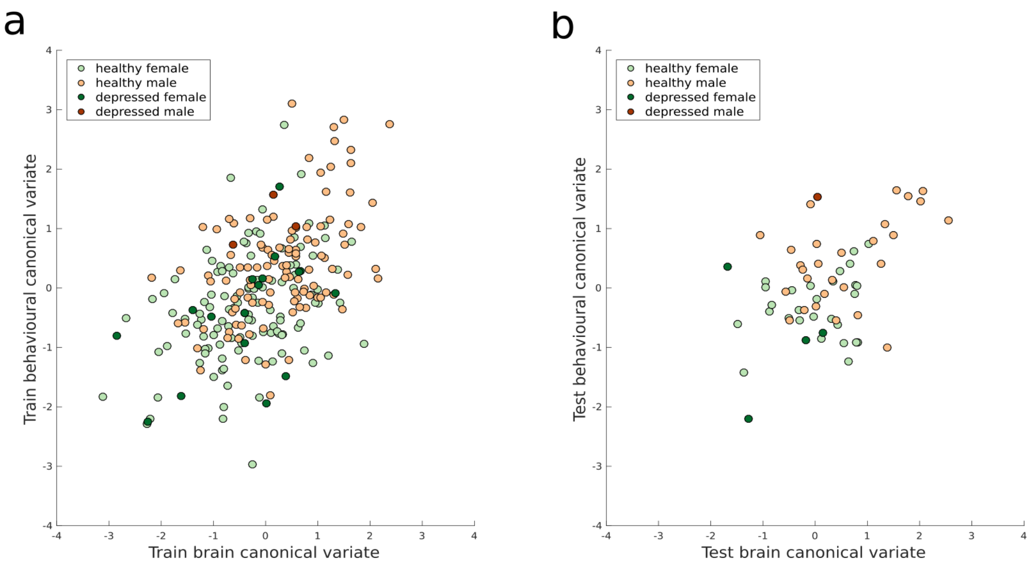

Figure S8: Significant brain-behaviour mode of population covariation for the training (**a**) and testing set (**b**) using the multiple hold-out framework. All the conventions are as in Supplementary Fig. S7.

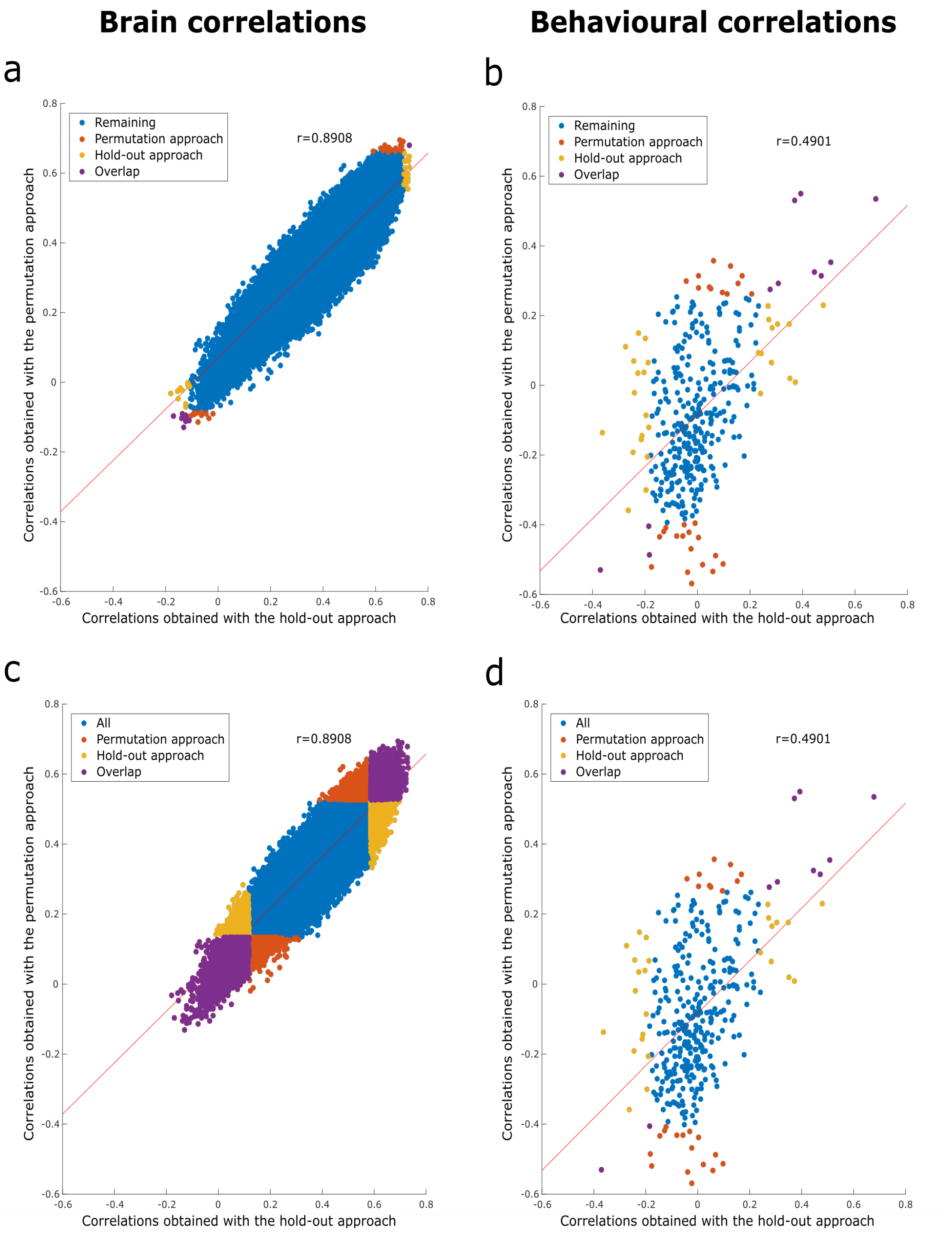

Figure S9: Scatter plots showing the brain (**a,c**) and behaviour (**b,d**) correlations with the first CCA mode obtained with both frameworks. (**a,b**) The overlap (purple) between the top 20 most positively/negatively correlated variables obtained with the permutation (orange) and multiple hold-out framework (yellow) is shown; (**c,d**) the same colour scheme is used to show the overlap between the top 5% most positively/negatively correlated variables obtained with the permutation and multiple hold-out framework. Blue denotes the remaining variables.

References

1. Kiddle, B. *et al.* Cohort Profile: The NSPN 2400 Cohort: a developmental sample supporting the Wellcome Trust NeuroScience in Psychiatry Network. *Int. J. Epidemiol.* **47,** 18–19g (2018).
2. Frick, P. & Hare, R. *The Antisocial Process Screening Device*. (Multi-Health Systems, 2001).
3. Patton, J. H., Stanford, M. S. & Barratt, E. S. Factor structure of the Barratt impulsiveness scale. *J. Clin. Psychol.* **51,** 768–74 (1995).
4. Lahey, B. B. *et al.* Psychometric characteristics of a measure of emotional dispositions developed to test a developmental propensity model of conduct disorder. *J. Clin. Child Adolesc. Psychol.* **37,** 794–807 (2008).
5. Fink, L. A., Bernstein, D., Handelsman, L., Foote, J. & Lovejoy, M. Initial reliability and validity of the childhood trauma interview: a new multidimensional measure of childhood interpersonal trauma. *Am. J. Psychiatry* **152,** 1329–35 (1995).
6. Essau, C. A., Sasagawa, S. & Frick, P. J. Callous-unemotional traits in a community sample of adolescents. *Assessment* **13,** 454–69 (2006).
7. Kessler, R. C. *et al.* Short screening scales to monitor population prevalences and trends in non-specific psychological distress. *Psychol. Med.* **32,** 959–76 (2002).
8. Bamber, D., Tamplin, A., Park, R. J., Kyte, Z. A. & Goodyer, I. M. Development of a short leyton obsessional inventory for children and adolescents. *J. Am. Acad. Child Adolesc. Psychiatry* **41,** 1246–52 (2002).
9. Costello, E. J. & Angold, A. Scales to assess child and adolescent depression: checklists, screens, and nets. *J. Am. Acad. Child Adolesc. Psychiatry* **27,** 726–737 (1988).
10. Reynolds, C. R. & Richmond, B. O. What I think and feel: a revised measure of Children's Manifest Anxiety. *J. Abnorm. Child Psychol.* **25,** 15–20 (1997).
11. Rosenberg, M. *Society and the adolescent self-image.* (Princeton University Press, 1965).
12. Raine, A. The SPQ: a scale for the assessment of schizotypal personality based on DSM-III-R criteria. *Schizophr. Bull.* **17,** 555–64 (1991).
13. Axelrod, B. N. Validity of the Wechsler abbreviated scale of intelligence and other very short forms of estimating intellectual functioning. *Assessment* **9,** 17–23 (2002).
14. Tennant, R. *et al.* The Warwick-Edinburgh Mental Well-being Scale (WEMWBS): development and UK validation. *Health Qual. Life Outcomes* **5,** 63 (2007).
15. Xia, M., Wang, J. & He, Y. BrainNet Viewer: A Network Visualization Tool for Human Brain Connectomics. *PLoS One* **8,** e68910 (2013).
16. Monteiro, J. M., Rao, A., Shawe-Taylor, J. & Mourão-Miranda, J. A multiple hold-out framework for Sparse Partial Least Squares. *J. Neurosci. Methods* **271,** 182–194 (2016).
17. Glasser, M. F. *et al.* A multi-modal parcellation of human cerebral cortex.

Nature **536,** 171–178 (2016).
